# Hierarchical Voting-Based Feature Selection and Ensemble Learning Model Scheme for Glioma Grading with Clinical and Molecular Characteristics

**DOI:** 10.3390/ijms232214155

**Published:** 2022-11-16

**Authors:** Erdal Tasci, Ying Zhuge, Harpreet Kaur, Kevin Camphausen, Andra Valentina Krauze

**Affiliations:** Radiation Oncology Branch, Center for Cancer Research, National Cancer Institute, National Institutes of Health, Building 10, Bethesda, MD 20892, USA

**Keywords:** diagnostic, brain tumor, glioma, grading, molecular data, oncology, machine learning, feature selection, pattern recognition

## Abstract

Determining the aggressiveness of gliomas, termed grading, is a critical step toward treatment optimization to increase the survival rate and decrease treatment toxicity for patients. Streamlined grading using molecular information has the potential to facilitate decision making in the clinic and aid in treatment planning. In recent years, molecular markers have increasingly gained importance in the classification of tumors. In this study, we propose a novel hierarchical voting-based methodology for improving the performance results of the feature selection stage and machine learning models for glioma grading with clinical and molecular predictors. To identify the best scheme for the given soft-voting-based ensemble learning model selections, we utilized publicly available TCGA and CGGA datasets and employed four dimensionality reduction methods to carry out a voting-based ensemble feature selection and five supervised models, with a total of sixteen combination sets. We also compared our proposed feature selection method with the LASSO feature selection method in isolation. The computational results indicate that the proposed method achieves 87.606% and 79.668% accuracy rates on TCGA and CGGA datasets, respectively, outperforming the LASSO feature selection method.

## 1. Introduction

Tumor grading, i.e., the ability to determine the biological aggressiveness of glioma, is very important for adequate management, including treatment planning and monitoring and the rate of survival in patients [1,2]. Gliomas are rapidly progressive, neurologically devastating, and the most common primary brain tumor originating from glial cells [3,4,5]. Standard-of-care treatment of gliomas is predicated on tumor grade, although it generally involves maximal surgical resection followed by radiation therapy (RT) with systemic treatment in the form of temozolomide (TMZ) chemotherapy administered concurrently and/or sequentially with the alternative of sequential PCV or PC (Procarbazine, CCNU with or without vincristine) [2,5]. According to the guidelines of the World Health Organization (WHO) classification of Central Nervous System (CNS) tumors, gliomas can be currently broadly categorized into two groups, low-grade gliomas (LGG) and high-grade gliomas (HGG), with glioblastoma multiforme (GBM), a high-grade glioma, based on histological and molecular parameters [6]. GBM is the most common, aggressive, invasive, and primary type of tumor. Glioma accounts for almost 80% of all primary malignant tumors of the brain, and GBM also accounts for more than 60% of all brain tumors in adults, emphasizing the importance of grading in this neuro-oncology scenario [7].

In recent years, as molecular alterations have gained importance for the classification of CNS tumors [6,8,9], a push for valued-added care has also added complexity to the discussion. Two facets emerged: the vast and heterogenous number of available molecular parameters emphasizing the importance of the identification and selection of the necessary molecular alterations, and the goal of reducing the cost of molecular testing to allow for its more widespread use and mitigation of health disparities. One example is the isocitrate dehydrogenase (IDH) mutation, a significant molecular marker to distinguish low-grade glioma from high-grade gliomas [10,11]. IDH testing has prompted discussion of cost and turnaround time, with USD 135 per p.R132H-specific IDH1 immunohistochemistry, USD 420 for single gene sequencing, and USD 1800 for next-generation sequencing [10]. Turnaround time utilized can take about two days for immunohistochemistry and 14 days for next-generation sequencing [10]. Clinical features such as age and gender [12] contribute to the tumor grading process, but there is a paucity of higher-level robust clinical annotation in publicly available datasets limiting links between pertinent molecular features and clinical data, which could advance value-added care as more widespread molecular testing may eventually benefit from the increase in reimbursement. Thus, selecting the best discriminative molecular and clinical markers not only reduces the cost to healthcare systems and patients while helping to curb growing health disparities in access to testing but also improves tumor grading performance, which can enable the selection of pertinent molecular features for future analyses and bench to bedside work as well as testing of novel targeted agents. The pattern recognition necessary to optimally leverage fragmented available molecular information is arguably not possible in the absence of computational analysis, hence our hypothesis that feature selection carries a significant role in this space.

Feature selection is concerned with the selection of the best feature subset from all features or patterns according to strategies that remove unrelated, insignificant, and redundant features [13]. Specifically, this process guarantees the best class prediction performance and reduces the computational demand/cost, increasing efficiency and providing more cost-effective features, increasing the classification accuracy rate, and improving the clarity of the results [14,15]. Today, feature selection is widely used in many data analysis applications, pattern recognition, and mining tasks [16,17]. As example studies of brain tumor grading, Ref. [18] performed subtyping and grading of lower-grade gliomas using integrated SVM recursive feature elimination and a correlation method using transcriptome data. In another study [19], molecular three-subtype classification of low-grade gliomas using magnetic-resonance-imaging-based radiomic features and employing genetic-algorithm-based feature selection with an eXtreme Gradient Boosting classifier was provided. In [20], a joint similarity network fusion (Joint-SNF) method was proposed to integrate different omic data types for subtype identification of Chinese lower-grade glioma. In [21], a deep-learning-based framework was developed for the precise and accurate classification of GBM subtypes by employing transcriptome and methylome data types. Although there is no single algorithm that could outperform every other machine learning model for all problems [22,23] (i.e., no free lunch theorem), many studies in the literature have shown that an ensemble of models generally outperforms individual models [22,24,25]. In recent years, ensemble learning methods have been considered state-of-the-art approaches for solving machine learning challenges [23,24,25,26,27]. In [28], the performances of advanced ensemble learning methods have been found to be more robust than the well-known Random Forest and AdaBoost ensemble classifiers. Currently, bagging and boosting models are one of the most popular ensemble learning methods, with Random Forest and AdaBoost being the most prominent and common implementations, respectively [29]. These methods cover a large scale of applications including face recognition, anomaly detection, and medicine [29]. The performance (e.g., the accuracy rate) of a machine learning model can be improved by training multiple learning models and combining their predictions (namely, classifier ensembles) [23,26,27,30,31]. This operation can be performed by employing various schemes such as bagging, boosting, or voting. Voting approaches can also be hard or soft voting based on the usage of the prediction results. Hard voting relies on only summing and majority voting the class outputs. Soft voting approaches yield more flexible and fine-grained results than hard majority voting due to handling probability scores for various machine learning tasks [32].

To this end, we aim to select the best subset of molecular and clinical features by reducing the number of features with various feature selection methods and ensemble-learning-based models in this study. We introduce a novel hierarchical voting-based methodology for the selection of the relevant features and improving the performance results of the machine learning models for the classification tasks. To reach the best performance result, we carried out experiments employing the possible combinations of supervised models in the soft-voting-based process employing two glioma datasets, TCGA and CGGA, that are currently in widespread use.

The main contributions of this study are summarized as follows:

- To the best of our knowledge, our study illustrates the first method that employs the hierarchical voting-based approach in the processes of both feature selection methods and ensemble learning models together to improve glioma grading results, which is an innovative approach to large-scale molecular glioma data.
- To the best of our knowledge, our study also constructs the first organized and structural dataset from TCGA LGG and GBM data that aggregates molecular and clinical information to allow for validation and data sharing and to speed up research progress for researchers working in this field.
- We focus on fusing the advantages of various feature selection methods and machine learning models via a voting-based procedure for glioma grading on two commonly used glioma datasets (TCGA and CGGA).
- We carry out comprehensive computational results for the comparison of our novel voting-based feature selection method with the LASSO feature selection method in isolation, given that these are two commonly employed glioma datasets that share similarities but also exhibit differences.
- We aim to identify the best-performing combination of a voting-based ensemble learning model for the feature selection stage to obtain the most accurate results given dataset variability in large-scale datasets with transferable use.
- The performances of voting-based ensemble models are evaluated with six measures for glioma grading.
- The extensive effects of the combinations of sixteen ensemble learning models are presented.

The remaining sections of this study are structured as follows: First, we briefly give an overview of the proposed methodology and explain the related feature selection methods and supervised learning models for glioma grading in Section 2. We describe the experimental procedures, datasets employed, and evaluation metrics and give the comprehensive experimental results of our method with discussions in Section 3. Finally, Section 4 contains the conclusion, discussion of the results, and possible future directions of this study.

## 2. Results

This section defines the experimental processes, the dataset used and its characteristics, and evaluation metrics. Then, we present the comprehensive experimental results of the performance metrics of tumor grading in the following subsections.

### 2.1. Experimental Process

In this study, the proposed methods were implemented in Python programming language with the scikit-learn [33] package for machine learning and the xverse [34] package for feature selection operations, and all the experiments were conducted on a system running a MacBook Pro laptop PC with macOS Monterey, 2.3 GHz 8-core Intel Core i9 CPU, and 16 GB 2667 MHz DDR4 RAM. The predictive models utilized are LR, SVM, KNN, RF, and AdaBoost. The combinations of 3, 4, and 5 of these models are used for the soft-voting-based ensemble learning scheme. The total number of ensemble learning model combinations is 16 (i.e., C(5,3) + C(5,4) + C(5,5) = 16). The corresponding combination set numbers and ensemble model combinations for the voting process are illustrated in Table 1.

As a preliminary study, we focused on decreasing the number of combinations of preprocessing stage by selecting appropriate normalization techniques from [0, 1] min-max normalization, [−1, 1] min-max normalization, or z-score normalization/standardization for age feature values. We experimented with these techniques on the various classifiers employed and decided to use the z-score normalization technique, given that it resulted in the best performance metric (i.e., accuracy rate) value obtained from the models.

For the minimum number of votes based feature selection, the weight of evidence, recursive feature elimination, Random Forest, and LASSO methods were applied. The minimum number of votes was set to 1 to eliminate features not chosen unanimously. For LASSO-based linear model operation, 10-fold cross-validation was carried out to find the best alpha parameter value through the iterations. A 10-fold cross-validation technique was also applied on the TCGA and CGGA datasets to construct and test the mean performance results of the learning models employed. In this study, the GBM class was considered positive and the LGG class was used as negative for the evaluation of learning models. The default values were assigned as the corresponding parameter values for the utilized classifiers (i.e., num of neighbors = 5, metric = ‘minkowski’ for KNN; C = 1, and kernel = ‘rbf’, gamma = ’scale’ for SVM; penalty = ‘l2’, *, dual = False, tol = 0.0001, C = 1.0, fit_intercept = True, intercept_scaling = 1, class_weight = None for LR; n_estimators = 100, *, criterion = ‘gini’, max_depth = None, min_samples_split = 2, min_samples_leaf = 1 for RF; n_estimators = 50, learning_rate = 1.0, algorithm = ‘SAMME.R’ for AdaBoost). We set the random state number to 0 for all employed learning models in order to obtain the same computational results with the same random state number on the datasets used.

### 2.2. Dataset

To evaluate our proposed methodology for the hierarchical voting-based processes, we utilized the two most widely employed genome atlas databases, namely The Cancer Genome Atlas (TCGA) and the Chinese Glioma Genome Atlas (CGGA) [35], for analyzing brain tumor (i.e., glioma) grading.

TCGA’s original dataset consists of 3 clinical features, the most frequently mutated 20 molecular/mutation features, and class labels for glioma grading, resulting in 23 total features (Table 2). The molecular features are captured as mutated or not_mutated (wildtype) depending on the TCGA Case_ID. The grades can be dichotomized into LGG (lower-grade glioma) or GBM (glioblastoma multiforme). The total numbers of instances for the TCGA dataset without and with preprocessing are 862 and 839, respectively. In the preprocessing stage, missing data from this dataset for gender, Age_at_diagnosis, or race were removed when feature values were captured as ‘--’, or ‘not reported’. Age_at_diagnosis feature values were also converted from string to continuous value by adding day information to the corresponding year information in the dataset as a floating-point number for the preprocessing stage. The CGGA dataset has 286 instances and 22 features (one less than TCGA) with the same characteristics illustrated in Table 2 except for the race feature, which is not directly specified in CGGA; however, CGGA documentation describes data origin as based on a Chinese cohort. There are no missing data for the CGGA dataset. The TCGA preprocessed dataset consists of 352 GBM and 487 LGG patients and there is also 102 GBM and 184 LGG patients for the CGGA dataset. No balancing/sampling strategy was applied for this study. The dataset query and storing operations were provided by NIDAP [36].

**Figure 1:**
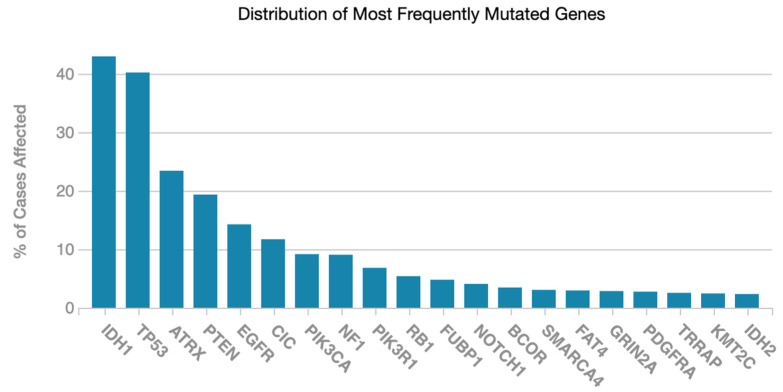
The distribution of the most frequently mutated genes for glioma data on TCGA.

According to TCGA, the distribution of the most frequently mutated 20 genes according to percentage of cases affected for glioma data are given in Figure 1. As shown in Figure 1, the percentage of cases affected decreases considerably after 20 molecular features, and computational load increases due to the number of features. Thus, we found it appropriate to choose 20 molecular features in this study.

### 2.3. Evaluation Metrics

To measure the tumor grading performance of the proposed hierarchical voting-based feature selection and ensemble learning methodology, we employed 6 evaluation metrics, classification accuracy rate (ACC), Area Under the ROC Curve (AUC), F-Measure (F1), precision (PRE), recall (REC), and specificity (SPEC) [37].

The classification accuracy rate was computed by dividing the total number of true positives and true negatives by the total number of true positives, false negatives, false positives, and true negatives. The equation is defined in Equation (1).

(1) $$\mathrm{ACC}=\frac{\mathrm{TP}+\mathrm{TN}}{\mathrm{TP}+\mathrm{TN}+\mathrm{FP}+\mathrm{FN}}$$

where TP, TN, FP, and FN represent the number of true positives, true negatives, false positives, and false negatives, respectively.

AUC, the Area Under the Receiver Operating Characteristic (ROC) curve, was constructed by plotting the true-positive rate against the false-positive rate for the performance of the binary learning model. The area of the maximum AUC value (i.e., 1) indicates a perfect test, and the AUC value of 0 specifies that the predictor classifies all instances incorrectly. A value of 0.5 for AUC indicates that the ROC curve will be diagonal (i.e., 45-degree line), and hence suggests that the diagnostic test has no discriminatory ability and prediction problem [38].

F-Measure is calculated by the harmonic mean of precision and recall. It is represented in Equation (2).

(2) $$F1=\frac{2\;\times \;\mathrm{PRE}\;\times \;\mathrm{REC}}{\mathrm{PRE}+\mathrm{REC}}$$

Precision means the positive predictive value. It is computed by dividing the number of true positives by the total number of true positives and false positives. The equation is illustrated in Equation (3).

(3) $$\mathrm{PRE}=\frac{\mathrm{TP}}{\mathrm{TP}+\mathrm{FP}}$$

Recall is the true-positive rate/hit rate or sensitivity. It is calculated by dividing the number of true positives by the total number of true positives and false negatives. The equation is shown in Equation (4).

(4) $$\mathrm{REC}=\frac{\mathrm{TP}}{\mathrm{TP}+\mathrm{FN}}$$

Specificity is expressed as the true negative rate. It is calculated by dividing the number of true negatives by the total number of false positives and true negatives. The equation of specificity is defined in Equation (5).

(5) $$\mathrm{SPEC}=\frac{\mathrm{TN}}{\mathrm{TN}+\mathrm{FP}}$$

### 2.4. Experimental Results

**Figure 2:**
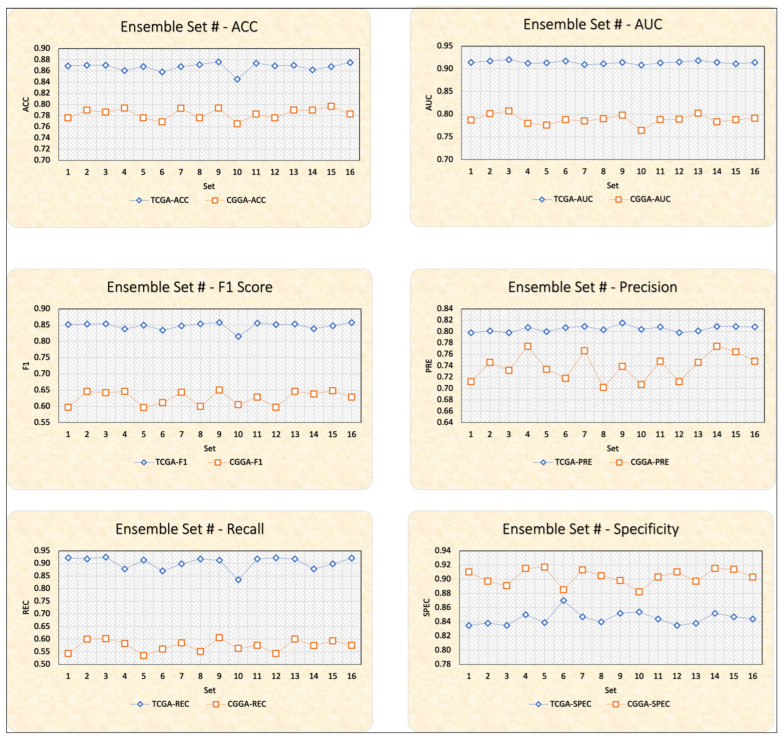
Graphical representations of the computational results for the TCGA and CGGA datasets.

In this study, we present the computational results to evaluate the effects of the features selection and ensemble learning model scheme stages for the TCGA and CGGA datasets (Figure 2 and Table 3, Table 4, Table 5, Table 6, Table 7, Table 8, Table 9, Table 10 and Table 11). Bold values indicate the best results.

We initially performed voting-based feature selection results using four methods (the weight of evidence, recursive feature elimination, Random Forest, and LASSO) for the ensemble combination sets in terms of ACC, AUC, F1, PRE, REC, and SPEC. Computational results revealed the best performance to originate from the ensemble of SVM, RF, and AdaBoost learning models with the values of 0.876, 0.858, and 0.815 for accuracy rate, F1, and precision values, respectively, for the TCGA dataset (Table 3) reflecting set combination #9 (Table 1) and the best accuracy rate value of 0.797 originating from the ensemble of SVM, KNN, RF, and AdaBoost for CGGA (Table 4) reflecting set combination #15 (Table 1).

The mean numbers of selected features after applying voting-based feature selection through 10-fold cross-validation on the TCGA and CGGA datasets are 14.9 and 17.6 for TCGA and CGGA, respectively (Table 5). The feature selection stage results in an approximate 35% and 20% cost gain in terms of feature reduction for the TCGA and CGGA datasets, respectively. A comprehensive comparison of the six performance metrics (ACC, AUC, F1, PRE, REC, and SPEC) on TCGA and CGGA datasets reveals CGGA’s superiority over TCGA in only one domain, that of specificity (Figure 2). A comparison of performance metrics with analysis carried out in the absence of the feature selection process reveals that the feature selection operation with the best ensemble combination yields approximately 1.38% accuracy rate improvement for the TCGA dataset as compared to CGGA (2.68%) (Table 4 with feature selection process vs. Table 6 without feature selection process).

We also investigated the effects of only using the LASSO feature selection method through individual supervised models on TCGA and CGGA datasets in comparison to the novel method, given the widespread use of LASSO in isolation (Table 7 and Table 8). The Logistic Regression classifier resulted in the best accuracy rate, AUC, precision, and specificity values with the values of 0.871, 0.920, 0.808, and 0.847, respectively, on the TCGA dataset, with the SVM model providing the best accuracy rate, AUC, precision, and specificity values (0.786, 0.817, 0.781, and 0.916), respectively, for CGGA.

Furthermore, we carried out experiments to observe the effects of only using the LASSO feature selection method through ensemble learning models on TCGA and CGGA datasets with the related combination sets (Table 9 and Table 10). The highest predictive performance was achieved with combination sets of models #2 and #13 (LR + SVM + RF and LR + SVM + RF + AdaBoost) with ACC 0.874 for TCGA (Table 9). These combination sets also resulted in the highest F-Measure result, with a value of 0.855. Since both combination sets resulted in the same results, we selected combination set #2, given its superior computational efficiency. The CGGA dataset combination set #9 (SVM + RF + AdaBoost) resulted in the highest predictive performance result, with an ACC of 0.786. Voting of SVM + RF + AdaBoost models also resulted in the highest F-Measure and recall results, with values of 0.648 and 0.594, respectively (Table 10).

When comparing the LASSO-only-based results with the voting-based feature selection results, we noted that the hierarchical voting-based feature selection and ensemble learning process provided more accurate results as compared to employing the LASSO feature selection method. The number of selected features when employing only the LASSO operation on TCGA and CGGA datasets (Table 11) resulted in a lower number of features than the total in both datasets but in particular in the CGGA set (11.9). The main advantage of the voting-based feature selection process is that it provides superior performance with cost improvement and gives the authorization to remove redundant features with unanimous selection.

## 3. Discussion

Based on the empirical results of our novel hierarchical voting-based feature selection and ensemble learning methodology including sixteen ensemble model combinations on two datasets with molecular and clinical characteristics, several insights follow.

Considering the predictive performance of the ensemble learning models utilized for the voting-based feature selection process in this study, our proposed feature selection method outperforms the results of the using only LASSO feature selection method. The following results were obtained:

- The empirical results indicate that our novel hierarchical voting-based feature selection and ensemble learning methodology can provide promising results for the glioma grading tasks. We employed four different feature selection methods and the combinations of five different individual learning models using the voting-based strategies to reach optimal results for this study. In this regard, to validate our ensemble feature selection method, we compared our methodology on two datasets with different ensemble model combinations and we outperformed the results of using the LASSO-only feature selection method.
- Our primary goal in this study was to reduce the cost and number of features of the proposed feature selection method and increase the prediction performance. In addition, although the selection of the best ensemble learning models and features varied in both datasets, we can conclude that our proposed novel ensemble-based feature selection method results in more accurate results as compared to only using the LASSO method. In this context, when datasets with a high sample number are obtained, factors such as the number of selected features, performance, and cost increase will become more stable.
- Our novel hierarchical voting-based methodology can be extended with a various number of feature selection methods and machine learning models as a framework to improve the many classifications, or pattern recognition tasks if desired.
- Voting-based ensemble schemes can give more flexibility as well as trustworthy and efficient results than those using individual schemes for related problems.
- Our feature selection methodology can also be combined with deep-learning-based feature reduction approaches.

The shortcomings of the dataset constructed are that we have a small size of data or features in this study since we accessed a limited number of important features; however, we need to explore higher-dimensional biomedical datasets to try these methods.

## 4. Materials and Methods

In this section, we present an overview of the proposed methodology for glioma grading and explain the feature selection and classification methods applied for this study. We also describe the ensemble learning process in the following subsections.

### 4.1. The Proposed Methodology for Glioma Grading

**Figure 3:**
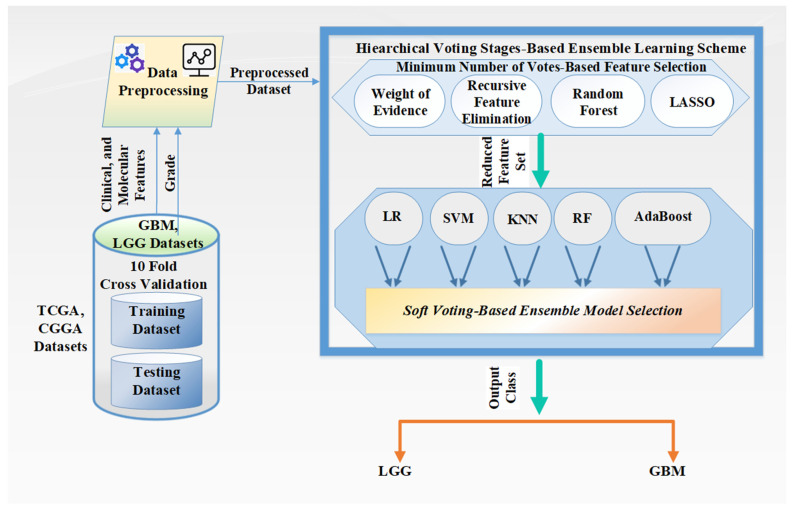
Overview of the proposed methodology.

The overview of the proposed methodology for glioma grading is illustrated in Figure 3. The hierarchical voting-based methodology includes two phases: (a) the minimum number of votes based feature selection and (b) soft-voting-based ensemble model selection.

As an initial step, the glioma datasets (TCGA and CGGA) are split into training–test datasets via a ten-fold cross-validation technique. Then, all clinical and molecular features and grade class label information are constructed for the subsequent machine-learning-related tasks. Next, data type conversion and removal of missing or insignificant values and columns are performed in the data preprocessing stage, followed by the hierarchical voting-stages-based ensemble learning scheme that is applied as a novel methodology for the glioma grading process. The substages of this methodology are briefly explained as follows.

The minimum number of votes based feature selection stage uses four different feature selection methods, weight of evidence, recursive feature elimination, Random Forest, and least absolute shrinkage and selection operator (LASSO), to rank features individually by employing the five supervised learning models according to their importance levels (e.g., if the importance level of the feature is equal to 0, it is disregarded). If the minimum number of votes is equal to 1, this means the feature to be selected must have at least a total of 1 vote from the four feature selection methods. Then, the selected features that meet the minimum number of votes following the administration of the four feature selection methods are transferred to the next level to experiment with all the possible combination sets for the voting-based ensemble learning process.

The soft-voting-based ensemble model selection stage consists of five supervised learning models (Logistic Regression, Support Vector Machine, K Nearest Neighbors, Random Forest, and AdaBoost). To merge the advantages of the individual learning models, we explored all three, four, and five combinations of all employed learning models to improve the classification performance results of the soft-voting-based ensemble learning models. Each method is explained in the following subsections in detail.

### 4.2. Feature Selection Methods

Feature selection methods are one of the types of dimensionality reduction approaches. They aim to find optimal feature subset n from all feature set m where n ≤ m [39]. These methods allow to improve the predictor performance, reduce the computational or memory cost, and facilitate data visualization by removing redundant and insignificant attributes in the pattern [40,41]. Feature selection methods often are categorized into three categories depending on if the learning model is utilized for the selection process as filter, wrapper, or embedded methods.

We explain the four feature selection methods (weight of evidence, recursive feature elimination, Random Forest, and LASSO) employed in this study in the following subsections.

#### 4.2.1. Weight of Evidence (WOE)

The weight of evidence is a quantitative method that estimates the predictive power of an independent variable in relation to the dependent variable in an easy way. The WOE method is a probabilistic approach based on a log-linear form of Bayesian rule [42]. WOE is also a helpful tool for identifying the relative risk based on the available information from the related pattern [43]. It is a type of data transformation technique that encodes categorical variables for classification. WOE handles outliers and helps to construct a linear relationship with log odds. The WOE method is defined in Equation (6) [44]. It is computed by taking the natural logarithm (ln) of the division of % of non-events and % of events.

(6) $$\mathrm{WOE}=\ln(\frac{\%\;\mathrm{of}\;\mathrm{non}\text{-}\mathrm{events}}{\%\;\mathrm{of}\;\mathrm{events}})$$

Given a hypothesis and some evidence, the WOE seeks to respond to the following question [45]: “How much does the evidence speak in favor of or against the hypothesis?” The WOE is usually defined for some evidence e, a hypothesis h, and its logical complement h. For example, in a simple binary classification setting, e = (X1,…, Xn), h: Y = 1, and h^−^: Y = 0.

As a concrete example [45], suppose that a doctor wants to know whether a patient’s symptoms indicate the presence of a certain disease, say, the flu. Denote e = “the patient has a fever,” h = “the patient has the flu,” and h^−^ = “the patient doesn’t have the flu.” The doctor might know that for a patient, the odds of having the flu roughly double once the patient’s fever is taken into account (i.e., the hypothesis-odds interpretation), which corresponds to WOE(h: e) ≈ log 2 [45]. Additional information about WOE can be obtained from [45].

#### 4.2.2. Recursive Feature Elimination (RFE)

The recursive feature elimination method is an embedded-based backward feature selection strategy [46,47]. RFE searches for a subset of features, starting with all features in the dataset and successfully removing features by fitting the specific machine learning algorithm until the specified number of features remains. This process is achieved by ranking the features in order of importance, discarding the least important features, and refitting the model through iterations. In this study, the Logistic Regression classifier is considered a specific machine learning model for the recursive feature elimination method in the feature selection stage.

#### 4.2.3. Random Forest (RF)

The Random Forest method selects features with respect to the importance values of features by ranking them. The importance of a feature is identified as how much this attribute is used in each tree of the forest. This is defined as the normalized total reduction in the metrics provided by that feature. In other words, important features are computed as the mean and standard deviation of accumulation of the impurity decrease within each tree [31].

#### 4.2.4. Least Absolute Shrinkage and Selection Operator (LASSO)

In statistics and machine learning, the least absolute shrinkage and selection operator is a linear model that estimates sparse coefficients of parameters. It is used for attribute selection, regularization, and regression analysis [48]. Mathematically, LASSO includes a linear model with an added regularization term. Suppose *y* = (*y*_1_,…, *y*_n_) T is the response vector and *x_j_* = (*x*_1*j*_,…, *x*_n*j*_) T, *j* = 1,…, *p*, are the linearly independent predictors. Let X = [*x*_1_,…, *x_p_*] be the predictor matrix. Assume the data are standardized. The LASSO estimates for the coefficients of a linear model are obtained by Equation (7) [49].

(7) $$\hat{\beta }=\underset{\beta }{arg\;min}\;\|y-\overset{p}{\underset{j=1}{\sum }}x_{j}\beta_{j}\|+\lambda \overset{p}{\underset{j=1}{\sum }}\left|\beta_{j}\right|$$

where *λ* is called the lasso regularization parameter and $\hat{\beta }$ is an exact unbiased estimate of the degrees of freedom of the lasso, and this result can be used to create an adaptive model selection metric for efficiently selecting the optimal LASSO fit [49]. The LASSO is a special case of the penalized least squares regression with an L1-penalty function, and if there is a high correlation in the feature groups, the LASSO method chooses only one among them and shrinks the coefficients of others to zero [50]. Thanks to this method, LASSO can contribute to both model accuracy and interpretability by removing irrelevant or insignificant features from the pattern.

### 4.3. Classification

In machine learning, the classification stage is a supervised learning approach that predicts corresponding class labels from the given set of examples/instances. Supervised learning algorithms use labeled datasets and outputs to train machine learning algorithms on how to predict a class label or outcome [51].

In this study, five supervised learning algorithms, namely Logistic Regression, Support Vector Machine, K Nearest Neighbors, Random Forest, and AdaBoost are employed for the machine learning tasks. We also applied a voting-based ensemble learning process to improve the classification results of these models. In the following subsections, we briefly describe these models.

#### 4.3.1. Logistic Regression

Logistic Regression is one of the simplest and most utilized statistical models for binary classification. It is a type of regression and predictive analysis technique that is used to explain the relationship between one dependent binary variable and one or more independent variables. The Logistic Regression model gives each variable a coefficient that measures its independent contribution to variation in the dependent variable [52].

#### 4.3.2. Support Vector Machine

Support Vector Machine is a supervised learning algorithm proposed by Vapnik et al. [53,54]. It originated from the structural risk minimization idea and is based on statistical learning theory [55]. For binary classification problems, input data are mapped to higher-dimensional space using various kernel structures. The SVM classifier explores and tries to find a hyperplane that has the maximum margin. This algorithm is very attractive, systematic, and effective for two-class and linear or non-linear classification problems [55].

#### 4.3.3. K Nearest Neighbors

K nearest neighbors is one of the most popular, simple, effective, robust, and fundamental classification models and lazy learning algorithms [56]. It categorizes test instances into the class of the closest instances based on distance measures and the number of neighbors. In other words, the test instance is assigned to the most common class among its k nearest neighbors by using the distance function that measures the difference or similarity between instances [4,56].

#### 4.3.4. Random Forest

Random Forest, proposed by Leo Breiman [57], is one of the most accurate, simple, easily parallelized, general-purpose machine learning predictor models [58,59]. It constructs an ensemble model with a set of decision trees that grow in randomly selected subspaces of data [58]. This bagging ensemble learning model is robust to outliers and noise and faster than bagging and boosting [59].

#### 4.3.5. AdaBoost

AdaBoost, short for Adaptive Boosting, is a type of statistical ensemble learning method. The output of the base learners is combined into a weighted sum that represents the final output of the boosted learning model. AdaBoost focuses on instances that were previously misclassified and, in each iteration, the weights of misclassified samples are increased while the weights of correctly classified samples are decreased [26].

### 4.4. Ensemble Learning

Ensemble learning methods are meta-approaches that combine the results of the multiple learning algorithms to achieve better prediction final performance results by using various schemes such as bagging, boosting, voting [27,32,60], or stacking. Voting approaches can be hard or soft based on their majority rule or predicted class probabilities. Soft voting approaches provide more well-calibrated, flexible, and fine-grained results than the hard-voting-based ensemble learning scheme [32,61]. In this study, soft-voting-based ensemble learning model combination sets are applied to obtain the best classification results with feature selection methods.

## 5. Conclusions

This study introduces a novel hierarchical voting-based feature selection and ensemble learning methodology for glioma grading. Our study uniquely explores ensemble feature selection methods and leverages ensemble learning models. Sixteen ensemble model combinations were employed to identify the best tumor grading performance on novel constructed datasets originating in the most widely employed molecular glioma databases, TCGA and CGGA, with captured molecular and clinical characteristics. Voting-based feature selection surpassed the results of using either the LASSO method or its combination with ensemble learning models on the TCGA and CGGA datasets. Considering the widespread use of LASSO and TCGA/CGGA, an optimized limit-free approach that confidently and consistently achieves superior performance is key. Our study illustrates that optimal feature selection and learning model selection are subject to both the number of instances and number of features in large-scale datasets, and that, given existing heterogeneity, ensemble approaches are beneficial for transferability and for obtaining superior results in healthcare datasets such as TCGA and CGGA, in spite of their trade-off strategy between computational workload and performance metrics (e.g., accuracy rate). We hope that our findings will serve as a basis for the development of efficient methods for value-added care pertaining to molecular markers while improving the predictive performance of models. The proposed methodology can also be utilized as a template framework to advance optimization considering the growing and already wide array of feature selection methods and machine learning models currently available for classification, pattern recognition, and data mining tasks.

As a future direction of this study, we plan to use additional biomedical datasets (clinical, imaging, and omic) with higher-dimensional features to both leverage and compare the performance of this method with other approaches. Additional or novel machine learning predictors, instead of the models in our study, can also be employed to analyze our methodology and potentially further improve the performance results for specific large-scale medical data scenarios.

## Figures and Tables

**Table 1:** The set numbers and the related learning model combinations for the voting process.

Set #	Model Combination
1	LR + SVM + KNN
2	LR + SVM + RF
3	LR + SVM + AdaBoost
4	LR + KNN + RF
5	LR + KNN + AdaBoost
6	LR + RF + AdaBoost
7	SVM + KNN + RF
8	SVM + KNN + AdaBoost
9	SVM + RF + AdaBoost
10	KNN + RF + AdaBoost
11	LR + SVM + KNN + RF
12	LR + SVM + KNN + AdaBoost
13	LR + SVM + RF + AdaBoost
14	LR + KNN + RF + AdaBoost
15	SVM + KNN + RF + AdaBoost
16	LR + SVM + KNN + RF + AdaBoost

**Table 2:** Features and class information for the datasets used. TCGA has 23 class features (3 clinical, 20 molecular), whereas CGGA has 22 given it is comprised of a Chinese population with race not explicitly specified in the database.

#	Type	Name	#	Type	Name	#	Type	Name
1	Clinical	Gender	9	Molecular	CIC	17	Molecular	BCOR
2	Clinical	Age_at_diagnosis	10	Molecular	MUC16	18	Molecular	CSMD3
3	Clinical	Race	11	Molecular	PIK3CA	19	Molecular	SMARCA4
4	Molecular	IDH1	12	Molecular	NF1	20	Molecular	GRIN2A
5	Molecular	TP53	13	Molecular	PIK3R1	21	Molecular	IDH2
6	Molecular	ATRX	14	Molecular	FUBP1	22	Molecular	FAT4
7	Molecular	PTEN	15	Molecular	RB1	23	Molecular	PDGFRA
8	Molecular	EGFR	16	Molecular	NOTCH1	24	Class	Grade

**Table 3:** Computational results for the TCGA dataset with the voting-based feature selection process.

Set #	ACC	AUC	F1	PRE	REC	SPEC
1	0.869	0.914	0.852	0.798	0.922	0.835
2	0.870	0.917	0.853	0.801	0.918	0.838
3	0.870	**0.920**	0.854	0.798	**0.925**	0.835
4	0.861	0.912	0.838	0.807	0.878	0.850
5	0.868	0.913	0.850	0.800	0.913	0.839
6	0.858	0.917	0.834	0.807	0.870	**0.870**
7	0.868	0.909	0.848	0.809	0.898	0.847
8	0.871	0.911	0.854	0.803	0.918	0.840
9	**0.876**	0.914	**0.858**	**0.815**	0.912	0.852
10	0.845	0.908	0.815	0.804	0.835	0.854
11	0.874	0.913	0.856	0.808	0.918	0.844
12	0.869	0.915	0.852	0.798	0.922	0.835
13	0.870	0.918	0.853	0.801	0.918	0.838
14	0.862	0.914	0.839	0.809	0.878	0.852
15	0.868	0.911	0.848	0.809	0.898	0.847
16	0.875	0.914	**0.858**	0.808	0.921	0.844

Bold values indicate the best results.

**Table 4:** Computational results for the CGGA dataset with the voting-based feature selection process.

Set #	ACC	AUC	F1	PRE	REC	SPEC
1	0.776	0.787	0.597	0.712	0.543	0.910
2	0.790	0.801	0.646	0.746	0.600	0.897
3	0.786	0.807	0.642	0.732	0.602	0.891
4	0.793	0.780	0.646	0.774	**0.583**	0.915
5	0.776	0.776	0.596	0.734	0.535	**0.917**
6	0.769	0.788	0.611	0.718	0.561	0.885
7	0.793	0.785	0.643	0.766	0.585	0.913
8	0.776	0.790	0.600	0.702	0.551	0.905
9	0.793	0.798	**0.650**	0.739	0.606	0.898
10	0.765	0.764	0.605	0.707	0.563	0.882
11	0.783	0.788	0.628	0.748	0.576	0.903
12	0.776	0.789	0.597	0.712	0.543	0.910
13	0.790	**0.802**	0.646	0.746	**0.600**	0.897
14	0.790	0.783	0.638	0.774	0.575	0.915
15	**0.797**	0.788	0.648	0.764	0.593	0.914
16	0.783	0.791	0.628	0.748	0.576	0.903

Bold values indicate the best results.

**Table 5:** The mean number of selected features after applying voting-based feature selection.

Dataset	TCGA	CGGA
Total # of Features	23	22
Selected # of Features	14.9	17.6

**Table 6:** The computational results without the feature selection process for the best ensemble set.

Dataset	Model	ACC	AUC	F1	PRE	REC	SPEC
TCGA	SVM + RF + AdaBoost	**0.864**	**0.914**	**0.842**	**0.807**	**0.885**	**0.850**
CGGA	SVM + KNN + RF + AdaBoost	**0.776**	0.804	**0.616**	**0.775**	**0.550**	**0.911**

Bold values indicate the best results.

**Table 7:** Computational results for TCGA dataset with LASSO feature selection method and individual models.

Model	ACC	AUC	F1	PRE	REC	SPEC
LR	**0.871**	**0.920**	0.853	**0.808**	0.911	**0.847**
SVM	**0.871**	0.909	**0.854**	0.801	**0.921**	0.838
KNN	0.839	0.901	0.809	0.787	0.838	0.840
RF	0.825	0.902	0.792	0.780	0.813	0.836
AdaBoost	0.863	0.914	0.842	0.803	0.895	0.845

Bold values indicate the best results.

**Table 8:** Computational results for CGGA dataset with LASSO feature selection method and individual models.

Model	ACC	AUC	F1	PRE	REC	SPEC
LR	0.780	0.793	**0.649**	0.730	**0.730**	0.887
SVM	**0.786**	**0.817**	0.639	**0.781**	0.566	**0.916**
KNN	0.744	0.752	0.555	0.682	0.497	0.888
RF	0.755	0.788	0.613	0.687	0.583	0.858
AdaBoost	0.748	0.772	0.600	0.653	0.572	0.849

Bold values indicate the best results.

**Table 9:** Computational results for TGGA dataset with LASSO feature selection method and ensemble learning models.

Set #	Voting of Model Combination	ACC	AUC	F1	PRE	REC	SPEC
1	LR + SVM + KNN	0.867	0.921	0.847	0.803	0.901	0.844
2	LR + SVM + RF	**0.874**	0.921	**0.855**	0.808	0.914	0.847
3	LR + SVM + AdaBoost	0.870	**0.923**	0.852	0.802	**0.915**	0.841
4	LR + KNN + RF	0.862	0.918	0.838	0.808	0.876	0.852
5	LR + KNN + AdaBoost	0.865	0.920	0.845	0.806	0.892	0.848
6	LR + RF + AdaBoost	0.858	0.921	0.834	0.807	0.870	0.853
7	SVM + KNN + RF	0.858	0.914	0.834	0.802	0.873	0.848
8	SVM + KNN + AdaBoost	0.867	0.916	0.846	0.808	0.892	0.851
9	SVM + RF + AdaBoost	0.867	0.914	0.844	0.809	0.887	0.853
10	KNN + RF + AdaBoost	0.846	0.909	0.816	0.802	0.837	**0.855**
11	LR + SVM + KNN + RF	0.863	0.919	0.840	0.804	0.885	0.848
12	LR + SVM + KNN + AdaBoost	0.868	0.921	0.849	0.806	0.901	0.846
13	LR + SVM + RF + AdaBoost	**0.874**	0.921	**0.855**	0.808	0.914	0.847
14	LR + KNN + RF + AdaBoost	0.863	0.918	0.839	**0.810**	0.876	0.854
15	SVM + KNN + RF + AdaBoost	0.861	0.914	0.837	0.805	0.876	0.850
16	LR + SVM + KNN + RF + AdaBoost	0.865	0.919	0.843	0.807	0.888	0.850

Bold values indicate the best results.

**Table 10:** Computational results for CGGA dataset with LASSO feature selection method and ensemble learning models.

Set #	Voting of Model Combination	ACC	AUC	F1	PRE	REC	SPEC
1	LR + SVM + KNN	0.783	0.806	0.636	**0.761**	0.567	**0.908**
2	LR + SVM + RF	0.779	**0.818**	0.637	0.752	0.577	0.897
3	LR + SVM + AdaBoost	0.776	0.810	0.637	0.739	0.587	0.884
4	LR + KNN + RF	0.779	0.805	0.629	0.749	0.568	0.903
5	LR + KNN + AdaBoost	0.772	0.798	0.619	0.736	0.560	0.897
6	LR + RF + AdaBoost	0.772	0.810	0.629	0.729	0.576	0.885
7	SVM + KNN + RF	0.768	0.803	0.619	0.722	0.570	0.887
8	SVM + KNN + AdaBoost	0.772	0.797	0.613	0.730	0.558	0.899
9	SVM + RF + AdaBoost	**0.786**	0.811	**0.648**	0.752	**0.594**	0.898
10	KNN + RF + AdaBoost	0.772	0.794	0.620	0.715	0.573	0.889
11	LR + SVM + KNN + RF	0.776	0.811	0.628	0.739	0.569	0.897
12	LR + SVM + KNN + AdaBoost	0.779	0.808	0.633	0.753	0.567	0.902
13	LR + SVM + RF + AdaBoost	0.779	0.816	0.637	0.752	0.578	0.897
14	LR + KNN + RF + AdaBoost	0.779	0.803	0.629	0.749	0.568	0.903
15	SVM + KNN + RF + AdaBoost	0.768	0.802	0.619	0.722	0.570	0.887
16	LR + SVM + KNN + RF + AdaBoost	0.776	0.811	0.628	0.739	0.569	0.897

Bold values indicate the best results.

**Table 11:** The mean number of selected features after applying LASSO feature selection.

Dataset	TCGA	CGGA
Total # of Features	23	22
Selected # of Features	20.5	11.9

## Data Availability

The data in this manuscript has been obtained from The Cancer Genome Atlas (TCGA) Research Network (https://www.cancer.gov/tcga) and the Chinese Glioma Genome Atlas (CGGA) (http://www.cgga.org.cn/).

## Abbreviations

ACC	Accuracy
AdaBoost	Adaptive Boosting
AUC	Area Under the ROC Curve
CGGA	Chinese Glioma Genome Atlas
CNS	Central Nervous System
F1	F-Measure
GBM	Glioblastoma Multiforme
HGG	High-Grade Glioma
IDH	Isocitrate Dehydrogenase
KNN	K Nearest Neighbors
LGG	Low-Grade Glioma
LASSO	Least Absolute Shrinkage and Selection Operator
LR	Logistic Regression
NCI	National Cancer Institute
NIH	National Institutes of Health
PRE	Precision
REC	Recall
RF	Random Forest
RT	Radiation Therapy
RFE	Recursive Feature Elimination
ROC	Receiver Operating Characteristics
SPEC	Specificity
SVM	Support Vector Machine
TCGA	The Cancer Genome Atlas
TMZ	Temozolomide
WHO	World Health Organization
WOE	Weight of Evidence
